# Morphology Formation in PC/ABS Blends during Thermal Processing and the Effect of the Viscosity Ratio of Blend Partners

**DOI:** 10.3390/ma9080659

**Published:** 2016-08-05

**Authors:** Stefanie Bärwinkel, Andreas Seidel, Sven Hobeika, Ralf Hufen, Michaela Mörl, Volker Altstädt

**Affiliations:** 1 Graduate School, University of Bayreuth, Universitätstraße 30, Bayreuth 95447, Germany; stefanie.baerwinkel@uni-bayreuth.de; 2 Covestro Deutschland AG, Business Unit Polycarbonates, Research & Development, Development Blends, Leverkusen 51365, Germany; andreas.seidel@covestro.com (A.S.); sven.hobeika@covestro.com (S.H.); ralf.hufen@covestro.com (R.H.); 3 Department of Polymer Engineering, University of Bayreuth, Universitätsstraße 30, Bayreuth 95447, Germany; michaela.kersch@uni-bayreuth.de

**Keywords:** polymer blend, skin-core morphology, blend morphology, viscosity ratio, PC/ABS, fracture surface

## Abstract

Morphology formation during compounding, as well as injection molding of blends containing 60 wt % polycarbonate (PC) and 40 wt % polybutadiene rubber-modified styrene-acrylonitrile copolymers (ABS), has been investigated by transmission electron microscopy (TEM). Profiles of the blend morphology have been recorded in injection-molded specimens and significant morphology gradients observed between their skin and core. A <10 µm thick surface layer with strongly dispersed and elongated nano-scale (streak-like) styrene acrylonitrile (SAN) phases and well-dispersed, isolated SAN-grafted polybutadiene rubber particles is followed by a 50–150 µm thick skin layer in which polymer morphology is characterized by lamellar SAN/ABS phases. Thickness of these lamellae increases with the distance from the specimen’s surface. In the core of the specimens the SAN-grafted polybutadiene rubber particles are exclusively present within the SAN phases, which exhibit a much coarser and less oriented, dispersed morphology compared to the skin. The effects of the viscosity of the SAN in the PC/ABS blends on phase morphologies and correlations with fracture mechanics in tensile and impact tests were investigated, including scanning electron microscopy (SEM) assessment of the fracture surfaces. A model explaining the mechanisms of morphology formation during injection molding of PC/ABS blends is discussed.

## 1. Introduction

The great industrial success of polycarbonate/polybutadiene rubber-modified styrene-acrylonitrile copolymers (PC/ABS) blends e.g., in the automotive sector is the result of the unique combination of the high heat distortion temperature of the PC, the good processability (melt flow) of the ABS, and a synergistic improvement of the low-temperature ductility. Suarez et al. studied the effect of the blend composition on the mechanical properties of PC/ABS blends [1]. They reported that PC/ABS blends show better properties than various other immiscible blend systems. Krache et al. described a synergistic effect for blends of PC and ABS in the ratio of 90/10, whereby the impact strength of the blend is even higher than the impact strength of the neat materials [2].

It is known that the mechanical properties of multiphase polymeric alloys, such as PC/ABS compounds, not only depend on the chemical nature and, thus, the inherent properties of the neat blend partners, but also on the phase morphology of the alloy [3]. A finely-dispersed phase morphology thereby typically results in improved ductility since external stresses can be more homogeneously dispersed throughout such materials, and the risk of initiation of failure due to local stress concentration is, thus, reduced [4].

The morphology of immiscible or partially miscible polymer blends depends on several factors, such as the rheological properties (viscosity ratio) of blend partners, blend composition, interfacial tension between blend partners, and processing conditions [5,6,7,8,9,10,11,12]. Taylor et al. and Grace et al. investigated the effect of viscosity ratio λ = η_d_/η_m_ on the size of the dispersed phase of immiscible fluids in general, where η_d_ is the viscosity of the dispersed phase and η_m_ the viscosity of the matrix phase [11,13]. It was found that, at a viscosity ratio close to one, the probability of getting a finely-dispersed droplet structure is high. The effect of the viscosity ratio of polymers in PC/ABS blends on the size of the dispersed phase resulting in a twin-screw compounding process was examined by Yang et al. [14]. Additionally, in this case the optimum dispersion (minimum phase domain size) of ABS in the PC was observed at a viscosity ratio of close to one. However, in commercial PC/ABS blends the viscosity ratio of ABS and PC is normally less than one, i.e., lower viscosity ABS is typically blended with PC in order to achieve a product with improved melt flow, but with still a high heat resistance [15]. For PC/SAN blends the effect of the compounding process, viscosity ratio, interfacial tension, and blend composition on the morphology resulting in the compounded pellet materials was studied by Wildes et al. [15]. The finest morphology was achieved when compounding was done with a twin-screw extruder. The smallest size of the dispersed phase in PC/SAN 70/30 blends containing SAN with 32.5 wt % acrylonitrile (AN) was found at a viscosity ratio η_SAN_/η_PC_ at around 0.35.

**Figure 1:**
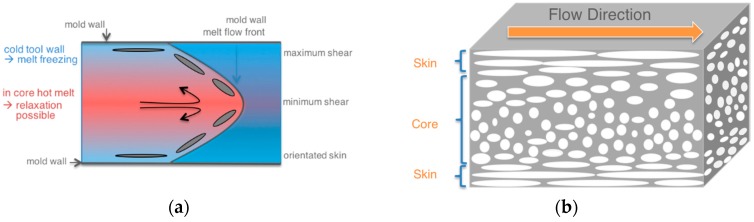
(**a**) Fountain flow experienced by polymers during injection molding of two-dimensional parts with a film gate (adapted from [17]) and (**b**) illustration of resulting profile of phase morphologies in the case of injection-molded parts made of blends of immiscible polymers.

Upon processing blends of immiscible polymers by injection molding, the melts experience shear force and temperature gradients that typically result in a hierarchical phase morphology profile often called “skin-core microstructure”. This hierarchical structure that is schematically shown in Figure 1b was first reported by Kato in 1968 on pure ABS materials [16]. In the skin layer of injection-molded parts, which in the literature for different polymer blend systems has been typically defined by a material layer within a depth of up to about 150 µm from the surface [17,18,19,20], the dispersed phase due to higher shear is undergoing strong deformation and eventually lamellae formation. In the core the melt experiences lower shear forces during injection molding and, obviously, a lower cooling rate due to the larger distance from the cold mold surface. This allows the dispersed phase to undergo relaxation, whereby a more dispersed droplet-like structure results and is eventually frozen [2,3,4,5].

The formation of the morphology in immiscible polymer blends during thermal processing thus results from different mechanistic processes, namely from phase deformation by shear and elongational stresses, relaxation, coalescence and, eventually, freezing. They are all highly dependent on the processing parameters, such as on melt and mold temperature, and injection speed. Extensive fundamental studies have been published on all of these aspects [20,21,22,23,24,25]. However, in the previously published investigations, the morphology in injection-molded PC/ABS blend systems was investigated only by scanning electron microscopy (SEM) within a depth from the surface of 130 µm [19], and so the real surface layer had not been reported in detail up until now in the literature.

Lee et al. examined the morphology of PC/ABS 30/70 [26] and PC/ABS 90/10 [19] blends by SEM investigation of the fracture surface after impact testing on injection-molded test specimens. In the PC-rich blends they observed near to the skin a bead-and-string formation of the ABS phases, which were oriented in the injection molding direction. In the core of the specimen, the ABS phase was seen as dispersed droplets, where rubber domains were located within the spherical particles of the SAN. It was proposed that the change of morphology in the core was due to break-up and end-pinching mechanisms caused by interfacial tension [19].

Due to the high industrial importance of PC/ABS blends, the macroscopic deformation and fracture toughness have already been repeatedly studied [27,28,29,30,31,32]. The fact that the morphology of such blends influences the mechanical properties was shown by Quintens et al., whereby the coarsening of the phase morphology was concluded to lead to a decreasing ductility [3]. Keitz et al. and Callaghan et al., reported that the optimal range of the AN content in the SAN in a PC/ABS blend in terms of optimization of material toughness was around 25–27 wt % [33,34]. So far, the effect of the SAN molecular weight and, thus, the SAN viscosity on the morphology and the corresponding mechanical properties of PC/ABS blends, however, had not yet been investigated.

The present study targets to investigate deeper into the formation of phase morphology during compounding and injection molding of PC/ABS 60/40 blends. Blends with this composition have a high industrial relevance as they exhibit a favorable balance of relatively high heat distortion temperature, good melt flow, and excellent low-temperature ductility, which fulfills the technical requirements of a wide variety of applications in the automotive sector. For the first time morphology formation will be assessed both in the real surface, the skin layer, and in the core of injection-molded parts. A particular focus of this study lies on the investigation of the molecular weight effect of the SAN in the ABS phase and, thus, of the viscosity ratio of the blend partners on the morphology formation in the PC/ABS blends as well as on its correlation with fracture mechanics of these materials.

## 2. Materials and Methods

### 2.1. Materials

Blends of a high molecular weight bisphenole-A (BPA)-based polycarbonate resin with emulsion-polymerized SAN-grafted particulate polybutadiene rubber (emulsion-ABS) and SAN resins of different molecular weight were compounded at 260 °C and 300 rpm in a co-rotating twin-screw extruder (ZSK M26 CC, Coperion GmbH, Stuttgart, Germany). Pellets were obtained by strand pelletization. The emulsion-ABS component contained 50 wt % of polybutadiene and a SAN-based grafting shell consisting of 28 wt % of acrylonitrile and 72 wt % of styrene. The 60 wt % of the SAN in this emulsion-ABS component was chemically grafted onto the surface of these rubber particles to improve their compatibility with both the SAN and PC polymers used as further blend constituents. The molecular weights (M_w_) of the SAN and PC resins and their polydispersity indices (D = M_w_/M_n_) describing the widths of the molecular weight distribution have been measured by gel permeation chromatography (GPC) with polystyrene- and BPA-based polycarbonate standards, respectively. Tetrahydrofurane (THF) and dichloromethane (DCM) have been used as both solvents and eluents for SAN and PC polymers, respectively, and poly(styrene-co-divinylbenzene) applied as a stationary phase in both cases. Refractory index detection was used for both kinds of polymers. GPC characterization data are summarized in Table 1. All three investigated SAN resins contained 24 wt % acrylonitrile and 76 wt % styrene, which leads to a partial miscibility with the PC [33,34]. The PC/ABS blend systems were compounded using 60 wt % of the polycarbonate, 20 wt % of the emulsion-ABS, and 20 wt % of the chosen SAN component (as shown also in Table 1).

Thus, the blend systems investigated in this study were as follows:

- 60% PC + 20% emulsion-ABS + 20% SAN low
- 60% PC + 20% emulsion-ABS + 20% SAN medium
- 60% PC + 20% emulsion-ABS + 20% SAN high

In the following the term “PC/ABS” will be used to designate the blends of PC, SAN, and emulsion-ABS. The term “ABS” will denote the mixtures of SAN and emulsion-ABS in the relative mass ratio of 50 wt %/50 wt % as used in the final PC/ABS compounds.

For the viscosity measurements on the neat ABS components compounds of 50 wt % of the emulsion-ABS component and 50 wt % of the respective SAN component, i.e., in the same mass ratio as used in the PC/ABS compounds, were pre-compounded at 260 °C and 300 rpm in a co-rotating twin-screw extruder (Coperion ZSK 26 MCC). The thus-obtained three ABS pre-compounds based on the high, medium, and low molecular weight SANs were denoted “ABS high”, “ABS medium”, and “ABS low”, respectively.

### 2.2. Methods

**Figure 2:**
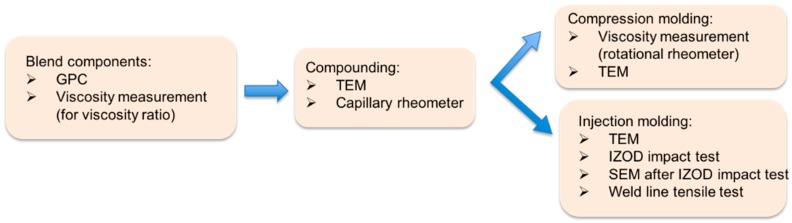
Processing scheme of this study.

Figure 2 illustrates the processing scheme and the characterizations, which were done in the different steps of this study.

#### 2.2.1. Injection Molding

Test bars used for mechanical testing and morphology characterization with TEM were produced by injection molding (on a HM110, Wittmann Battenfeld GmbH & Co. KG, Meinerzhagen, Germany) with a melt temperature of 260 °C, a mold temperature of 80 °C, and an injection speed of 40 mm/s. These are typical injection molding conditions used in the PC/ABS industry to mold test bars for characterization of mechanical material properties. In injection molding of more complex and larger real applications parts different, specifically adjusted molding parameters, e.g., higher injection speeds and melt temperatures, may be typically required. The effect of variation of molding conditions on morphology formation in PC/ABS blends is, however, not within the scope of this investigation, but will be treated in another future study.

#### 2.2.2. Compression Molding

For parallel plate shear rheology the pellets were compression molded with a hot press at 260 °C. The processing conditions were: preheating 4 min without pressure, then 4 min under 22 bar and cooling to RT in another press for 1–2 min at 9 bar.

#### 2.2.3. TEM

**Figure 3:**
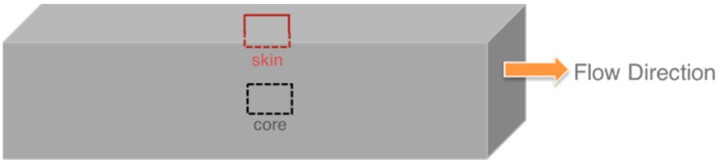
Illustration of injection-molded specimen with marked cutting positions for TEM investigations.

The morphology was characterized by transmission electron microscopy (TEM) both within the compounded pellets and the injection- and compression-molded test bars. For investigation of the morphology, the pellets obtained from the compounding process were cut in different directions relative to the extrusion direction with an ultramicrotome (Leica EM UC7, Leica Mikrosysteme GmbH, Wetzlar, Germany) with a thickness around 50 nm. The TEM cut was prepared from the middle of the compression-molded specimen. Eventually, for each compound sample, the injection-molded notched Izod impact test bars with a geometry of 80 mm × 10 mm × 4 mm were cut at half length (i.e., at a distance of 40 mm from the injection gate position) parallel to the injection molding direction, both at a position in the core (around 2 mm from surface) and directly in the surface of the specimens (see Figure 3). Panorama views of the morphology profile in the skin phase of the injection-molded specimens were recorded by assembling a series of 20% overlapping TEM pictures taken on a single cut that had been collected at the surface of the injection-molded specimens. Between each of these overlapping TEM pictures the cut was slightly shifted along the axis perpendicular to the melt flow direction during injection molding.

The specimens were first stained with OsO_4_ for 30 s in vacuum to make the polybutadiene (PB) in the ABS component appear black. Afterwards the PC phase was stained with RuO_4_ for 15 min, which makes the matrix phase appear grey in the TEM pictures. The morphology was observed by transmission electron microscopy (TEM Leo EM922Omega, Carl Zeiss AG, Oberkochen, Germany).

#### 2.2.4. Viscosity Measurements by Rotational and Capillary Rheometer

The melt viscosities at lower shear rates from 0.1 to 500 rad/s were measured using the compression-molded specimens via rotational rheometer with parallel plate geometry (RDAIII Rheometric Scientific) at 260 °C and 10% strain (at this strain the material is still in the linear elastic region, which was proven by the strain sweep measurements of the material). With a capillary rheometer the viscosity was measured at 260 °C according to ISO 11443-A over the shear rate range from 50 to 1500 s^−1^. In this case the compounded pellets were used as test specimens.

#### 2.2.5. Impact Strength

The PC/ABS blends were injection-molded at 260 °C melt temperature, 80 °C mold temperature and 40 mm/s injection speed to provide test bars of dimension 80 mm × 10 mm × 4.0 mm. The impact strength of notched specimens was measured on these test bars according to Izod test DIN EN ISO 180/1A at −30 °C. For calculation of standard deviations ten impact experiments were performed on each compound.

#### 2.2.6. SEM

After Izod impact testing at −30 °C the fractured surfaces of the samples were analyzed by scanning electron microscopy (SEM Leo Gemini 1530 for higher magnifications (Carl Zeiss AG, Oberkochen, Germany), SEM JSM6510 for lower magnification (Jeol GmbH, Freising, Germany). The samples were sputtered with a layer of platinum.

#### 2.2.7. Weld Line Tensile Strength

The PC/ABS blends were injection-molded at 260 °C melt temperature, 80 °C mold temperature, and 40 mm/s injection speed using a double gate mold to provide tensile bars with dimensions of 170 mm × 10 mm × 4.0 mm with a weld line. The tensile strength examined with these specimens having a weld-line is defined as the weld line tensile strength. The weld line tensile strength was measured with a universal testing machine (Zwick Z020) (Zwick GmbH & Co. KG, Ulm, Germany) according to DIN EN ISO 527-1 with a testing speed of 50 mm/min at 23 °C. For the calculation of standard deviation, five weld line tensile bars of each compound were assessed.

## 3. Results and Discussion

### 3.1. Rheological Properties of the Blend Components and the Blend Systems

**Figure 4:**
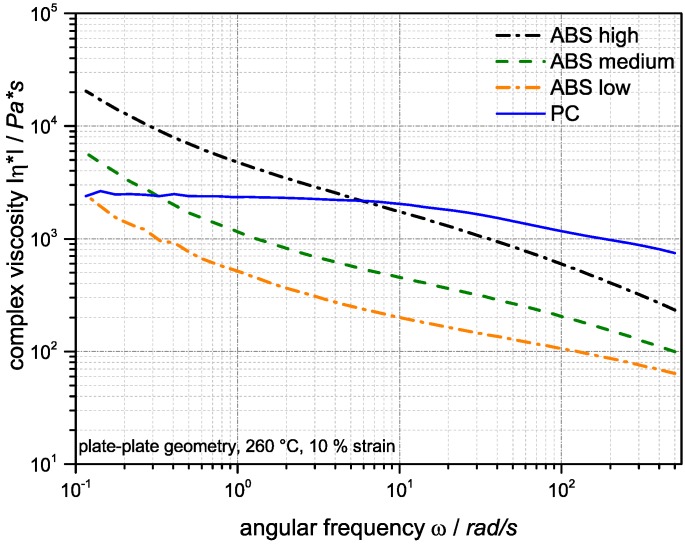
Shear rate dependence of the complex viscosity measured at 260 °C of the neat PC and ABS components used for build-up of the investigated PC/ABS blends.

Figure 4 shows the complex viscosity of the neat polycarbonate and ABS components used in the built-up of the investigated PC/ABS blends as function of the oscillatory frequency (or shear rate) at a temperature of 260 °C, which is the processing temperature of the blend systems during its compounding, as well as injection molding.

The ABS components show a shear thinning behavior and, as expected, an increase of melt viscosity with the molecular weight of the SAN components. The increase of ABS viscosities at lower shear rates is a consequence of the network formation of the dispersed polybutadiene rubber particles [35]. The viscosity of the PC shows a nearly Newtonian behavior over a wide frequency range. At the higher shear rates of >50 rad/s typically occurring during compounding in a twin-screw extruder and during injection molding, the viscosity of the PC component is higher compared to the viscosities of all three investigated ABS components. On the other hand, due to the network formation of the butadiene particles in the ABS and the mostly Newtonian behavior of the PC, at lower shear rates the melt viscosities of the ABS become higher compared to the PC. The frequency, at which the viscosities of the PC and ABS are equal, obviously increases with increasing molecular weight of the SAN in the ABS.

The effect of the ratio of viscosities of immiscible or partly miscible polymeric blend partners on the resulting phase morphology of polymer blend systems has been investigated in the literature [14,36,37,38,39]. During compounding and injection molding different shear rates are known to occur. For further discussions of morphology formation, the ratios of the viscosities of the dispersed phase (ABS) and the matrix phase (PC) were calculated at the lower and higher shear rates approximating, to our best knowledge, the different shear conditions occurring during compounding and injection molding processing, respectively. For compounding in a twin-screw extruder, typically shear rates in the order of magnitude of 100 s^−1^ are described in the literature [40]. For injection molding, shear rates depend on injection speeds and other parameters, and generally are assumed to be in the order of magnitude of >1000 s^−1^. The calculation of the viscosity ratios λ_d/m_ = λ_ABS/PC_ experienced by the blend during its compounding and injection molding was thus done on the basis of the viscosities of the ABS and the PC as measured by plate-plate rheometer (at 100 rad/s) and at the highest shear rate that was possible to measure with the available equipment for all blend components by a capillary rheometer (i.e., at 1500 s^−1^), respectively (see Table 2 and Table 3). The values of the viscosity ratios at 100 rad/s (Table 2) show an increasing viscosity ratio from 0.11 to 0.40 with increasing molecular weight of the SAN.

The viscosity ratios at 1500 s^−1^ (Table 3) also increase with increasing molecular weight of the SAN from 0.11 to 0.18. Thus, the trend in the viscosity ratios for the investigated samples at the lower and higher shear rates experienced by the blend during compounding and injection molding, respectively, are, in principle, the same. However, while the viscosity ratio values calculated for higher and lower shear rates are practically the same for the PC/ABS blends based on the low and medium molecular weight SANs, for the blend based on the high molecular weight SAN the viscosity ratio turns out to be much lower in the case of the higher shear frequency. Hence, under the shear conditions typical of injection molding, the viscosity ratios of the investigated blend partners covers only a relatively small range, although the molecular weights of the SAN polymers covers the complete range that is commercially available.

**Figure 5:**
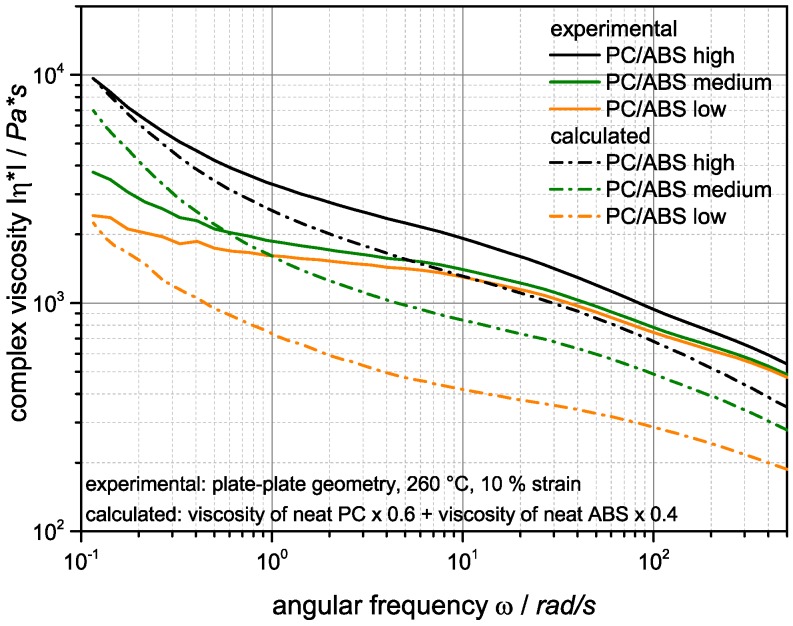
Shear rate dependence of the complex viscosities measured (solid lines) at 260 °C for the investigated PC/ABS blends containing SAN of different molecular weight (high, medium, and low). The dotted lines show a comparison with the weight-averaged viscosities of the blends components PC and the respective ABS, calculated as 0.6 × η_PC_ + 0.4 × η_ABS_.

The complex melt viscosities of the investigated PC/ABS 60/40 blends as a function of angular frequency are shown in Figure 5. The shear viscosity of all PC/ABS blends is decreasing with increasing frequency, which is characteristic for a shear thinning behavior.

The PC/ABS curves of all three PC/ABS blend samples have nearly the same shape and, as expected, are just shifted towards lower melt viscosities with decreasing molecular weight of the SAN component in the blend. The rheological behavior of the blend systems is determined by both blend partners and shows a synergetic reduction of the viscosity vs. the calculated weight averaged viscosities of the blend partners PC and ABS in the majority of the assessed angular frequency range, in particular at higher shear rates typical of melt processes, such as compounding and injection molding. At very low shear rates below 0.1–1 rad/s the shear thinning behavior of the ABS however becomes dominant.

### 3.2. Morphology Formation during Compounding

During compounding of the PC/ABS blends, starting from the individual polymers PC, emulsion-ABS and SAN, the formation of blend morphology depends on the shear conditions, the viscosity ratio of the blend partners and, in principle, also their interfacial tension. In this study interfacial tension can be regarded as constant for all investigated PC/ABS samples as always the same PC and emulsion-ABS components are used and all SAN polymers have the same monomer composition.

**Figure 6:**
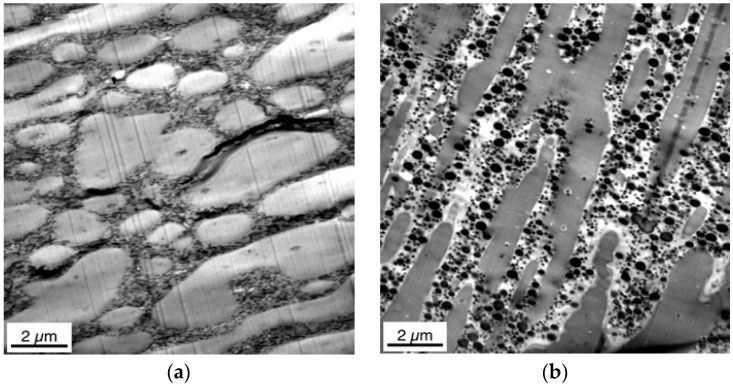
TEM micrographs of compounded pellets of PC/ABS blend with SAN medium. (**a**,**b**) cuts with different orientations relative towards the strand extrusion direction.

TEM micrographs taken on ultramicrotome cuts that had been collected from the compounded pellets in different orientations relative towards the strand extrusion direction, show a severely different morphology characterized by a different degree of deformation and orientation of the ABS phase (Figure 6). Interestingly, depending on the cutting orientation, morphology in the compounded pellets is either phase-inverted versus the expectation for a PC/ABS 60/40 blend, i.e., ABS forms the matrix and PC forms the dispersed phase (Figure 6a) or is co-continuous (Figure 6b).

Obviously, for interpretation of TEM micrographs on pellets it is crucial to provide detailed information about the position and direction of the investigated microtome cut. In published TEM studies on pellets, where this information has not been explicitly provided, the interpretations and drawn conclusions have to be taken with high caution, in particular since it is very difficult to define and, thus, to keep constant the cutting orientation in compounded pellets of a series of samples.

**Figure 7:**
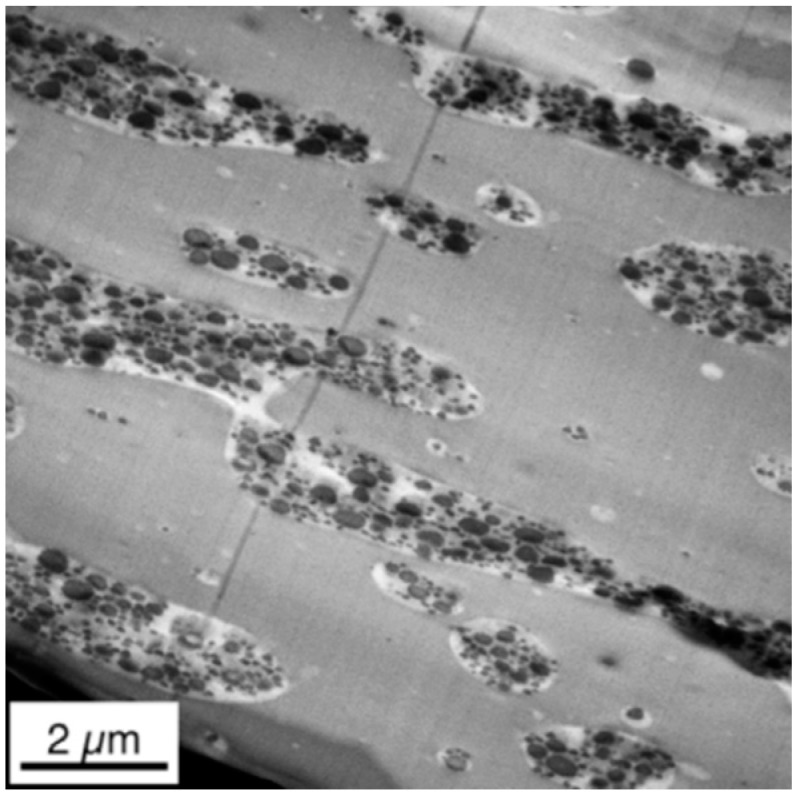
TEM micrograph of compression-molded sample of PC/ABS blend with SAN medium cut from the middle of the specimen.

The morphology of the compressed sample shows a similar morphology compared to that of the pellet. This is shown in Figure 7. Due to no significant effect of compression molding on the morphology of PC/ABS, the micrograph will not be further discussed.

### 3.3. Morphology Formation during Injection Molding

During injection molding of a thermoplastic material the melt is experiencing a combination of shear and elongational flow field gradients. In blends of immiscible or partly miscible polymers this results in different phase morphologies at different positions in the specimen. Another consequence are different morphology views depending on the orientation of the microtome cut relative to the orientation of the melt flow in the injection molding process at the position where the cut is taken.

In this study, the phase morphology of the injection-molded specimens was always investigated on microtome cuts generated in a direction parallel to the injection flow, as shown in Figure 3.

The morphology of polymer blends is preferentially investigated by TEM as it allows good visual contrast separation of the different polymer phases. A major disadvantage of TEM versus SEM images is that TEM can only visualize a very small section of the specimen. So far published TEM investigations of the skin section of injection-molded specimens have commonly studied a layer covering the first 150 µm from the surface of the specimens. Only a very small part of this large layer was actually visualized by the published TEM pictures and a more comprehensive view of the real surface was not shown. Hence, these previous studies left the question where, within the 150 µm layer, the particular picture had been taken, i.e., these studies were implicitly suggesting a homogeneous morphology within this 150 µm thick skin layer.

**Figure 8:**
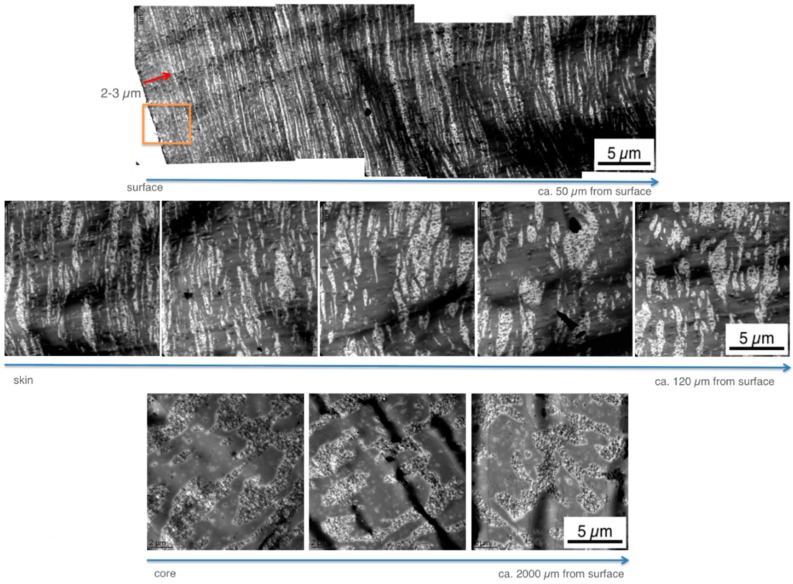
TEM micrographs visualizing the phase morphology profiles within the real surface (first 50 µm layer—upper row), the morphology gradients within the skin layer (first 120 µm from the surface—middle row) and the morphology within the core (bottom row) of an injection-molded specimen made of the PC/ABS blend based on low molecular weight SAN. The orange box in the upper row panorama view illustrates the image detail that is shown at higher magnifications in Figure 8.

**Figure 9:**
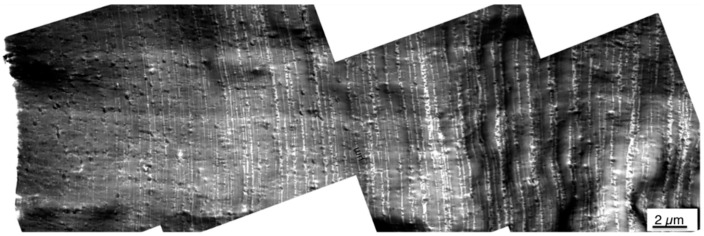
TEM micrographs visualizing the first 50 µm surface layer morphology profile of an injection-molded specimen made of the PC/ABS blend based on high molecular weight SAN.

In contrast to the previous work, in the present study the microtome cuts have been prepared at the surface itself and the TEM pictures have been taken in such a way to explicitly show this surface. Thus, it is always well-defined at which depth below the surface a certain morphology is observed. Moreover, morphology profiles were recorded within the skin layers of the injection-molded specimens prepared from the PC/ABS blends. Figure 8 and Figure 9 show skin morphology profile examples for the PC/ABS blends based on low and high molecular weight SAN polymers, respectively. Panorama views of the first 50 µm surface layer are shown in the top row of Figure 8 and Figure 9. The middle row of Figure 6 shows non-overlapping TEM micrographs taken at different distances from the surface up to a depth of about 120 µm, still within the skin layer of the injection-molded specimen. All of the micrographs shown in the top and middle row of Figure 8 were taken on a single microtome cut generated in the skin of the specimen as shown in Figure 3. In addition, pictures were taken on various microtome cuts generated in the core of the injection-molded specimens, i.e., at a distance of about 2000 µm below their surface (see bottom row in Figure 6). Hence, Figure 6 shows the full profile from the surface up to the core in the very center of the injection-molded specimen. Thus, for the first time, the true surface and the morphology changes appearing from skin to core of the specimen are visualized. On the left side of the upper row micrograph in Figure 8 and Figure 9, where the white backgrounds start, the real surfaces of the test specimens are visible. The morphology within the first 5 µm surface layer of the specimen made of the PC/ABS blend based on the low molecular weight SAN (image detail marked by the orange box in the upper row panorama view of Figure 8) is shown in Figure 8 at higher magnifications to visualize the very special microscopic morphology here, which is described in the present investigation for the first time.

The panorama view in Figure 9 for the PC/ABS blend based on the high molecular weight SAN shows the same specimen surface section that is visualized in the upper row of Figure 8 for the respective material based on the low molecular weight SAN.

**Figure 10:**
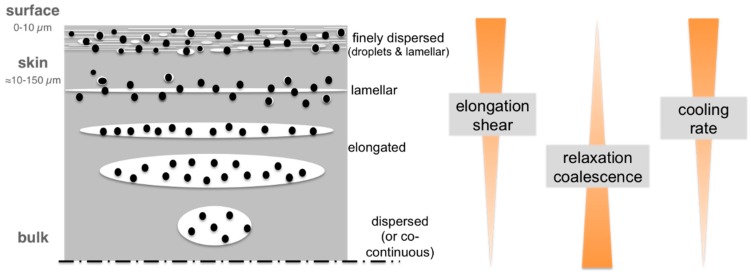
Illustration of morphology formation during injection molding of PC/ABS blends.

On the basis of this morphology profile, and the already published data [17,18,19,20], a more precise and distinguished model of the PC/ABS blend morphology formation during injection molding was developed and is displayed in Figure 10.

**Figure 11:**
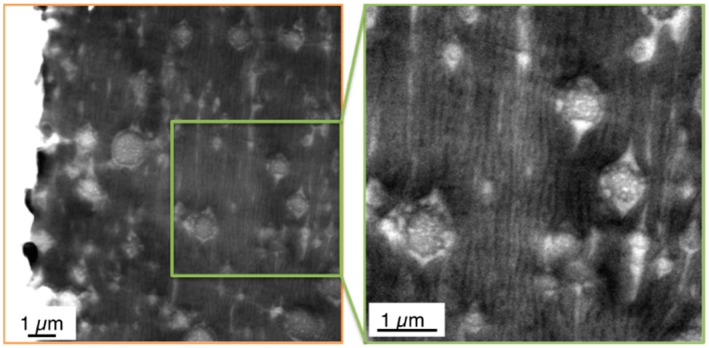
TEM micrograph visualizing the phase morphology in the surface (first 5 µm layer) of an injection-molded specimen made of the PC/ABS blend based on SAN with low molecular weight SAN at two different magnifications. The zoom-in picture on the right hand side shows the streak-like elongated SAN phases (which appear black at this thickness of only around 10–20 nm).

Within the specimens injection-molded from the currently investigated PC/ABS 60/40 compositions, in the core the ABS domains are relatively coarsely dispersed, only slightly deformed, or form a co-continuous network with the polycarbonate. Due to the reduced cooling rate in the core compared to the skin layer, thermodynamically unstable phase deformations resulting from the shear and elongational flow fields had obviously sufficient possibility to relax and provided ABS domains time for some coalescence. The morphology observed in the core, thus, can be assumed to be closer to the thermodynamic equilibrium state compared to the morphology observed nearer to the surface. With decreasing distance from the surface the dispersed ABS phases experience higher shear and elongational forces upon melt injection and, thus, are more strongly stretched. At the same time the cooling rate increases with decreasing distance from the cold mold surface and, thus, time for relaxation of the stretched domains is restricted, i.e., the non-equilibrium, strongly oriented phase morphology is partly frozen. With further decreasing distance from the surface these deformation processes become more pronounced and eventually result in lamellar ABS morphologies. In the core morphologies described so far, the SAN-grafted polybutadiene rubber particles are exclusively found in the SAN domains. The thickness of the ABS lamellae decreases with decreasing distance from the surface of the specimen. While due to their SAN grafting shell the rubber particles preferred to be dispersed in the SAN phase in the core, this is not any more possible when deformation of the SAN domains eventually result in a decrease of the thickness of these lamellae below the diameter of the rubber particles (100–400 nm). This happens very near to the surface (within a 5–50 µm layer). In the PC/ABS blend based on low molecular weight SAN the thickness of the lamellae in the first 15 µm from the surface e.g., ranges from 50 and 300 nm. Here the SAN-grafted rubber particles increasingly become dispersed in the polycarbonate phase leaving pure SAN lamellae, which are now free of rubber particles. These rubber-free, very low-viscosity SAN lamellae are even more susceptible to deformation. Thus, under the effect of the very high shear and elongational forces related to the friction close to the mold surface, and due to the missing elasticity originally provoked by the rubber phase, they cannot withstand any more break up into finely-dispersed and strongly-elongated streak-like SAN nano-phases, with a thickness in the range of only 10–20 nm. These can only be visualized at the highest TEM magnifications (see Figure 11b).

**Figure 12:**
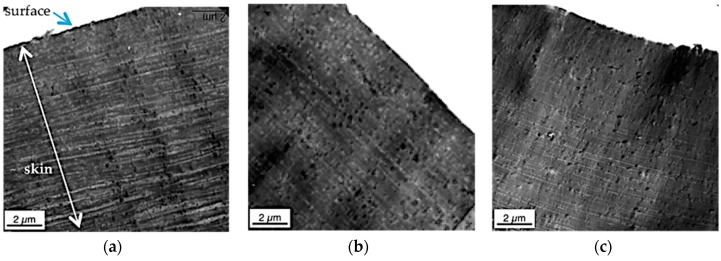
TEM micrographs of the skin layer (0–12 µm from the surface) taken from specimens injection-molded from PC/ABS blends with (**a**) SAN low; (**b**) SAN medium; and (**c**) SAN high.

Isolated polybutadiene rubber particles surrounded by a thin SAN grafting shell are left behind in the PC phase. This special surface morphology is observed only within the first few microns from the real surface of the specimens, because practically instantaneous freezing does not allow any coalescence of this energetically unfavorable, non-equilibrium state there. In principle, the same hierarchical morphology profile was observed in the injection-molded specimens of all three PC/ABS blends independent on the molecular weight of the SAN polymer (compare upper row of Figure 8 with Figure 9 and see Figure 12 comparing the surface morphology of all three investigated PC/ABS specimens).

The thickness of the surface layer, which is characterized by the strongly dispersed and elongated (streak like) SAN nano-phases, is increasing with increasing molecular weight of the SAN from 2 to 3 µm in case of the blend based on the SAN with low molecular weight to 5–6 µm for the blend based on the SAN with high molecular weight. The thickness of the ABS lamellae, which are characteristic of the skin layer following the direct surface layer, decreases with increasing molecular weight of the SAN. In the specimen made of the PC/ABS blend based on low molecular weight SAN these lamellae are up to 300 nm thick at a depth of 12 µm below the specimen’s surface, whereas in the specimen made of the blend based on the high molecular weight SAN maximum lamellar thickness at this depth is only 50 nm. Moreover, the thickness of the skin layer in which the ABS/SAN phases exhibit a lamellar morphology increases with the viscosity of the SAN in the PC/ABS blend. While in the specimen made of the PC/ABS containing low molecular weight SAN the transition from lamellar to elongated dispersed phase morphology starts already within a distance of about 25 µm from the surface (see upper row in Figure 8), in the specimen of the PC/ABS based on the high molecular weight SAN the thickness of the lamellar skin layer is >40 µm (see Figure 8). The reason for all three observations is believed to be the higher shear forces resulting during injection molding from the viscosity increase of the melt due to the molecular weight increase of the SAN polymer component (see also Figure 4 and Table 3).

**Figure 13:**
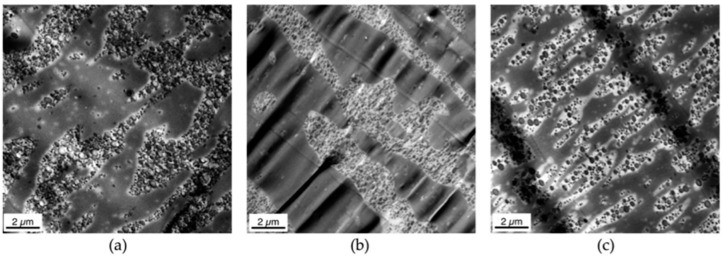
TEM micrographs of the core of specimens injection-molded from PC/ABS blends with (**a**) SAN low; (**b**) SAN medium; and (**c**) SAN high.

Figure 13 shows the TEM micrographs recorded on microtome cuts generated from the core of the different injection-molded specimens, i.e., at a distance of about 2000 µm below the surface. The core phase morphologies of all three investigated PC/ABS blend specimens differing in molecular weight of the SAN polymer constituent are very similar to each other, but they are strongly different from those previously described morphologies observed for the same materials at the surface and in the skin layers of the injection-molded specimens (compare Figure 12 with Figure 13).

The ABS domains in the core of injection-molded PC/ABS specimens in all different specimens are only slightly oriented by injection molding compared to the morphology observed in the skin. The morphologies of the PC/ABS blends with SAN low and SAN medium look practically identical. This observation correlates with the similar viscosity ratio λ_ABS/PC_ within the two blend systems (0.11 for the blend based on SAN low and 0.13 for the blend based on SAN medium, measured at the high shear rates typical of injection molding). Slightly larger ABS domains are visible in the bulk of these two blends compared to the PC/ABS blend with high molecular weight SAN and, thus, with a significantly, but not drastically, higher viscosity ratio λ_d/m_ of 0.18, measured at a shear rate of 1500 s^−1^ (see also Table 3). In the PC/ABS blend based on the high molecular weight SAN, the ABS domains show increased deformation, smaller domain sizes, and are obviously in a transition to break up. We believe that this is caused by the higher viscosity ratio of λ_d/m_ favoring improved dispersion and, consequently, smaller phase domain sizes as was already concluded in the previously published literature [11,12,14]. Additionally, due to the higher melt viscosity of the blend based on the high molecular weight SAN (see Table 2 and Figure 4), the shear forces experienced by the melt upon injection molding are increased, so phase deformation as a necessary precursor of drop break up can be assumed to be more pronounced. Furthermore, the higher molecular weight of the SAN also prevents phase coalescence of the dispersed ABS phase as it increases its viscosity.

### 3.4. Correlations of Core Morpholgy with Mechanical Properties

**Figure 14:**
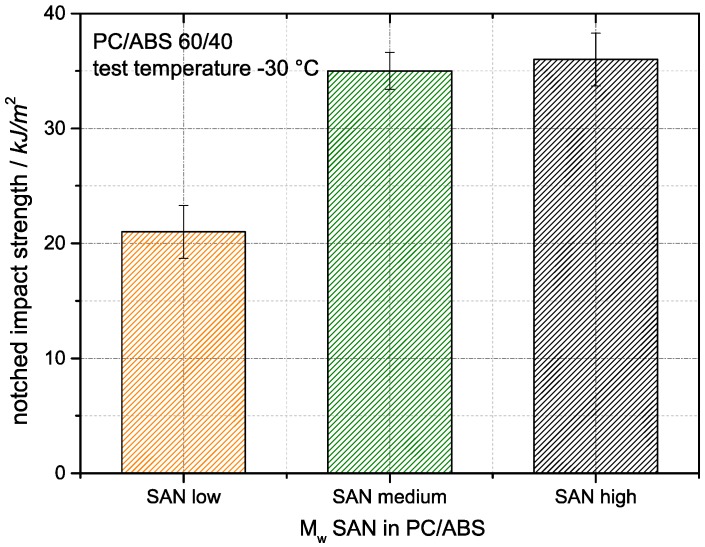
Effect of SAN molecular weight on the impact strength of PC/ABS blends at −30 °C.

Figure 14 shows the Izod notched impact strengths of the three PC/ABS blends differing in molecular weight of the SAN polymers that have been measured at −30 °C. The blends based on the medium and high molecular weight SAN show the same impact strength levels within the accuracy of the measurement. Obviously, beyond a certain threshold limit of SAN molecular weight (above 50,000 but below 100,000 g/mol) the impact strength of PC/ABS blend materials of this particular composition is not sensitive any more towards variations of the SAN molecular weight. On the other hand, the PC/ABS blend based on the lowest molecular weight SAN polymer shows a significantly lower Izod notched impact strength even though SAN low has a much lower polydispersity of molecular weight compared to SAN medium and high. Typically the mechanical properties of polymers improve with decreasing polydispersity due to the lower fraction of lowest molecular weight contributors [41]. Therefore, for a SAN with the same Mw as SAN low but with the same D as for SAN medium and SAN high, an even lower Izod notched impact strength would be expected. According to general experience, Izod notched impact strength levels >>30 kJ/m^2^ are known to typically correspond to a ductile failure while values significantly below this value are indicative of a rather brittle performance. Hence, the PC/ABS blends based on the medium and high molecular weight SAN were expected to show an essentially ductile failure, while the blend based on the SAN with the lowest molecular weight obviously was expected to show a clearly brittle behavior at low temperature. The decreasing Izod notched impact strength observed with the PC/ABS blend with the lowest molecular weight SAN is believed to be a consequence of the lower inherent fracture toughness of the low molecular weight SAN blend constituent. Morphology can be excluded as a major root cause for the ductility differences since the observed core morphologies were identical for the blends based on the low and medium molecular weight SANs (see Figure 13a,b), which are different in low-temperature impact strength, while the materials based on the medium and high molecular weight SANs show different core morphologies (see Figure 13b,c) but exhibit practically the same low-temperature impact strength.

**Figure 15:**
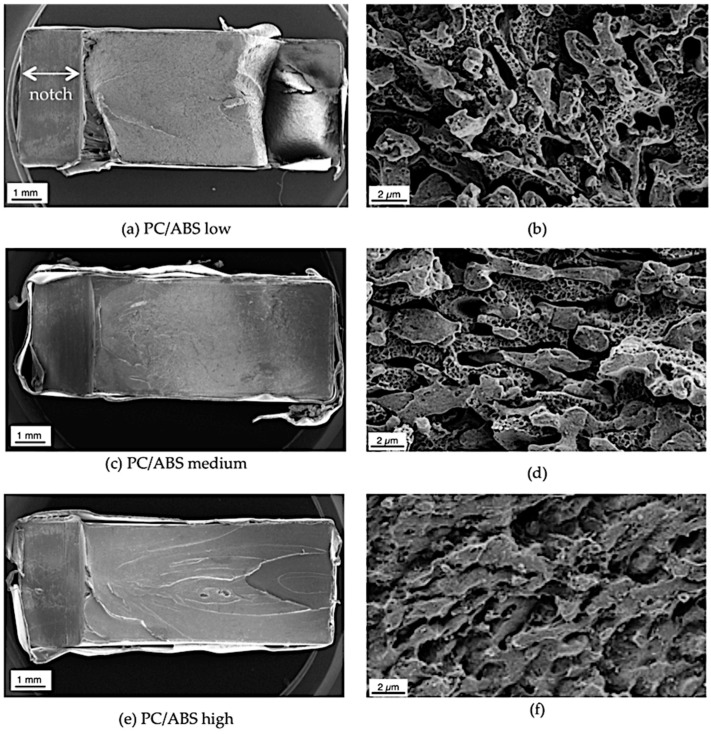
SEM micrographs of fracture surfaces of PC/ABS blends after Izod impact testing at −30 °C: Blends based on (**a**,**b**) SAN low; (**c**,**d**) SAN medium; and (**e**,**f**) SAN high. The notch with a length of around 1.5 mm can be seen on the left side of the SEM pictures taken at the lower magnification.

The fracture surfaces of the Izod impact strength specimens after testing at −30 °C were investigated via SEM to confirm the mode of impact failure. SEM micrographs are shown in Figure 15 at a low magnification visualizing overviews of the complete fracture surface and thus the crack propagation path and at a higher magnification at a position in the middle of the crack path to visualize the phase adhesion during impact failure.

The overview SEM micrographs show that the fracture surface smoothness increases with decreasing molecular weight of the SAN. In the case of the blend based on the low molecular weight SAN, the material shows a failure with material delamination. These results confirm an increasing ductility of the PC/ABS blend with increasing molecular weight of the SAN in good correlation with the measured values of the Izod notched impact strengths (Figure 14). The SEM pictures at higher magnification for the PC/ABS blends based on the low and medium molecular weight SANs (Figure 15b,d) compared to the material based on the high molecular weight SAN (Figure 15f) show a more pronounced detachment of the polybutadiene rubber particles from the SAN (little voids at the size of the rubber particles) and also a more pronounced detachment of the ABS from the PC phases (smoother phase surfaces) during impact failure. Interfacial tension between the ABS and the PC phases has to be assumed to be the same for each of the three PC/ABS samples differing only in molecular weight of the SAN but not in the chemical nature and composition of the polymeric blend partners. Nevertheless, SEM micrographs give a strong indication that interphase adhesion is increasing with the SAN molecular weight. The reason is believed to be a stronger intermolecular entanglement of SAN and PC molecules in the interphase with increasing SAN molecular weight. Such entanglement obviously requires a SAN molecular weight threshold above 100,000 g/mol to be exceeded in order to become effective in enabling stress transfer between polymer phases to such an extent that a ductile fracture pattern results. Although a good interphase adhesion between PC and ABS domains resulting from intermolecular entanglement obviously seems to be a requirement to achieve a fully ductile low-temperature notched impact failure pattern, the actually measured Izod notched impact strength values seem to be less affected by this interphase adhesion, but rather predominantly determined by the inherent mechanical strength of the individual polymeric blend partners. Sufficient hetero-molecular entanglement between SAN and PC molecules needed for good interphase adhesion and good homo-molecular entanglement of SAN molecules required for sufficient inherent SAN toughness are found to require exceedance of different SAN molecular weight thresholds. Ductile low-temperature notched impact failure in a PC/ABS blend of the investigated composition requires that SAN molecular weight exceeds a threshold in the range of 100,000–170,000 g/mol, whereas high low-temperature Izod notched impact strength values require that SAN molecular weight exceeds only a lower threshold in the range of 50,000–100,000 g/mol.

**Figure 16:**
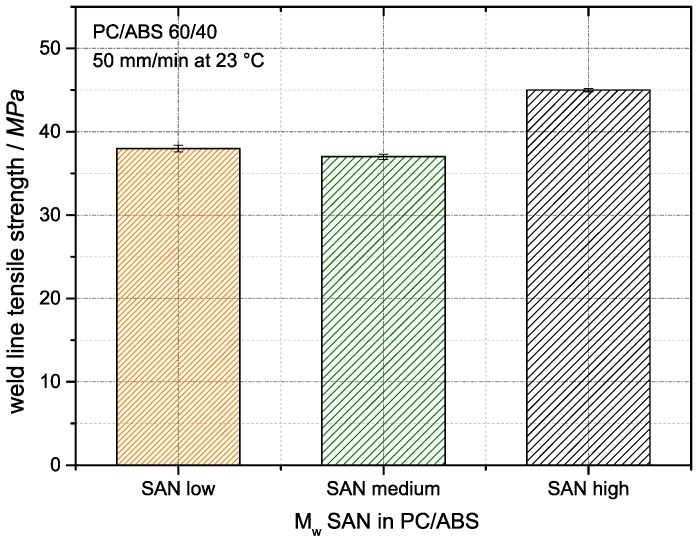
Effect of SAN molecular weight on the weld line tensile strength of PC/ABS blends at 23 °C.

The conclusions from the SEM micrographs concerning the interphase adhesion between PC and ABS domains is independent of the molecular weight of the SAN are confirmed by the results of weld line tensile tests, which are displayed in Figure 16. The weld line strength has been concluded in the literature to be a good indicator for changes of phase compatibility or phase adhesion, respectively, in heterogeneous polymer blends [42,43].

The weld line strength of the currently investigated PC/ABS blends based on low and medium molecular weight SANs are identical within the accuracy of measurement (37–38 MPa), whereas the weld line strength of the PC/ABS compound based on the high molecular weight SAN with a value of 45 MPa is 20% higher. These results correlate with the behavior seen in the SEM micrographs in Figure 15, where the fracture surfaces of the PC/ABS blends with the low and medium molecular weight SAN showed the same morphology indicative of severe phase detachment upon impact testing, while the SEM micrograph of the material based on the high molecular weight SAN suggested a significantly different (improved) phase adhesion.

## 4. Conclusions

In this study of PC/ABS 60/40 blends it has been shown that morphology of the materials strongly depends on the processing history of the investigated specimen and also on the viewing angle, relative to the melt flow direction applied during this processing. Hence, since with compounded pellets it is hardly possible to define the orientation of a microtome cut for microscopic investigations, conclusions on morphological differences observed between different pellet samples have to be taken with high caution. Moreover, morphologies in compounded pellets and injection-molded specimens have been shown to display significant differences.

During injection molding of the PC/ABS blends different morphologies are demonstrated to be formed in the surface, the skin, and the core of a specimen. A mechanistic model assuming a combination of processes, namely:

(a) phase deformation by shear and elongational forces, which decrease with the distance from the mold surface;
(b) relaxation and phase coalescence increasing with the distance from the surface because of the decreasing local cooling rate; and
(c) freezing of non-equilibrium phase morphologies, which is faster nearer to the cold mold surface, can explain the morphology profiles observed in injection-molded parts made of PC/ABS blends.

The result is a <10 µm thick surface layer with strongly dispersed and elongated (streak-like) SAN phases exhibiting a thickness of 10–20 nm and with isolated, SAN-grafted polybutadiene rubber particles. This surface layer is followed by a 25 to >50 µm thick skin layer, in which polymer morphology is characterized by lamellar SAN/ABS phases. The thickness of these lamellae increases with the distance from the specimen’s surface. Within the first 15 µm from the surface, the lamellae thickness is typically in the range of 50 to 300 nm. In contrast to the skin layer, in the core of the specimens the SAN grafted polybutadiene rubber particles are exclusively present within the SAN phase. The rubber containing SAN phases in the core show a coarser, only slightly oriented dispersed or co-continuous morphology with the PC.

Phase morphologies in the core of injection-molded specimens, in principle, turn out to be only slightly affected by the molecular weight of the SAN in the blend. Only the thicknesses of the surface and skin layers, as well as the thickness of the SAN/ABS lamellae, show a dependence on the SAN molecular weight. This is likely related to the increasing melt viscosities of the PC/ABS blends with increasing SAN molecular weight resulting in higher shear and, thus, in increased ABS phase deformation.

Izod notched impact strengths of PC/ABS blends of the investigated composition turn out to be mostly independent of the molecular weight of the SAN above a threshold level, which is in the range >>50,000 and <100,000 g/mol, but the values decrease significantly below this threshold SAN molecular weight. SEM micrographs of the fracture surface after Izod notched impact testing and weld line tensile strength measurements suggest an increasing phase adhesion between PC and ABS domains with an increasing molecular weight of SAN above a different threshold level, which is in the range of >100,000 g/mol and <170,000 g/mol. This is likely caused by increased intermolecular entanglements of PC and SAN molecules in the interphase above this SAN molecular weight. While the mode of fracture (ductile/brittle failure) observed in Izod notched impact testing at −30 °C turns out to be correlated with this interphase adhesion, the actually measured Izod notched impact strength values are predominantly correlated with the inherent strength of the polymeric blend partners rather than related to the interphase interactions and to phase morphology.

The improved understanding developed in this study of the morphology formation in PC/ABS blends during compounding and thermal processing, and of the morphology-properties correlations in such systems, is believed to be, in principle, transferable to other blends of immiscible or partly-miscible polymers and to enable, on a mid-term perspective, tailoring of such blends for new industrial applications.

## Figures and Tables

**Table 1:** Properties of blend components.

Sample	M_w_ in g/mol	D	Amount in Composition in %	Supplier
PC	31,000	2.5	60	Covestro
emulsion-ABS			20	Elix Polymers
SAN high	170,000	13	20	Styrolution
SAN medium	102,000	13	20	Styrolution
SAN low	51,000	2.8	20	UMG

**Table 2:** Calculation of viscosity ratios λ_d/m_ = λ_ABS/PC_ at an angular frequency of 100 rad/s measured with a rotational rheometer with parallel plate geometry.

	SAN Low	PC/ABS SAN Medium	SAN High
η_PC_ (Pa*s)	1554	1554	1554
η_ABS_ (Pa*s)	171	210	618
λ_d/m_	0.11	0.14	0.40

**Table 3:** Calculation of viscosity ratios λ_d/m_ = λ_ABS/PC_ at a shear rate of 1500 s**^−^**^1^ measured with a capillary rheometer.

	SAN Low	PC/ABS SAN Medium	SAN High
η_PC_ (Pa*s)	667	667	667
η_ABS_ (Pa*s)	72	89	122
λ_d/m_	0.11	0.13	0.18
